# A “Cookbook” for Vulnerability Research

**DOI:** 10.3389/fpubh.2019.00352

**Published:** 2019-11-21

**Authors:** Paula S. Tallman, Armando Valdés-Velásquez, Gabriela Salmón-Mulanovich, Gwenyth O. Lee, Amy R. Riley-Powell, Luciana Blanco-Villafuerte, Stella M. Hartinger, Valerie A. Paz-Soldán

**Affiliations:** ^1^Keller Science Action Department, The Field Museum of Natural History, Chicago, IL, United States; ^2^School of Sciences and Philosophy, Universidad Peruana Cayetano Heredia, Lima, Peru; ^3^School of Public Health and Administration, Universidad Peruana Cayetano Heredia, Lima, Peru; ^4^Department of Biomedical Engineering PUCP-UPCH, Pontificia Universidad Católica del Perú, Lima, Peru; ^5^School of Public Health and Tropical Medicine, Tulane University, New Orleans, LA, United States; ^6^School of Public Health, University of Michigan, Ann Arbor, MI, United States; ^7^Institute of Development Studies, University of Sussex, Sussex, United Kingdom; ^8^The Swiss Tropical and Public Health Institute, University of Basel, Basel, Switzerland

**Keywords:** vulnerability, health, socio-ecological systems, place-based, comparative, theory, methods

## Abstract

There is a growing need to facilitate the interdisciplinary study of the relationship between the environment and human health and well-being. It is increasingly recognized that vulnerability is a key construct allowing discipline-specific research questions on these topics to be meaningfully contextualized. However, there is little consensus regarding the meaning of the concept of vulnerability or how it can best be utilized in research studies. In this perspective article, we use the metaphor of a “cookbook” to review promising trends in vulnerability research and to make this body of research accessible to a multi-disciplinary audience. Specifically, we discuss a selection of “recipes” (theoretical frameworks), “ingredients” (vulnerability domains), “cooking tools” (qualitative and quantitative methods), and approaches to “meal presentation” (communication of results) drawn from vulnerability studies published in the past 15 years. Our aim is for this short “cookbook” to serve as a jumping-off point for scholars unfamiliar with the vulnerability literature and an inspiration for scholars more familiar with this topic to develop new ways to navigate the tension between locally-specific assessments of vulnerability and attempts at standardization. Our ultimate take-home message is that the specifics theories and methods used in vulnerability research are less important than attention to what we see as the 3 ‘T’s of *transparency, triangulation*, and *transferability*, and to efforts to make vulnerability research both “place-based” and comparable.

## Introduction

Multiple disciplines, including public health, anthropology, economics, and ecology, concern themselves with the health, well-being, and livelihoods of marginalized communities (1–3). It is increasingly recognized that vulnerability is a key construct allowing discipline-specific research questions on these topics to be meaningfully contextualized. Thus, the concept of vulnerability can build bridges for cross-disciplinary communication and research (4) and potentially provide a mechanism for connecting science and policy (5). However, there is little consensus on “best practices” in vulnerability research (6) and obstacles remain in developing measures that are applicable across different contexts (7). Still, scholars interested in vulnerability aim to advance approaches that facilitate comparability (8), while remaining attentive to the fact that vulnerability is place-based and “inescapably contextual” [(9), p. 508].

With these goals and limitations in mind we present the beginnings of a “cookbook” for interdisciplinary vulnerability research that highlights the 3 ‘T's of *transparency, triangulation, and transferability*. Extending the metaphor of the cookbook in the first section of this article we discuss four commonly used theoretical approaches in vulnerability research (different “recipes”). We do not present a one-size fits all solution but review these theories to encourage researchers to be *transparent* in the framing of their work and to aid in pinpointing a number of domains (“ingredients”) for vulnerability assessments, discussed in the second section. In the third section, we pinpoint methods (“cooking tools”), which can measure these domains and use a few short examples that highlight the *triangulation* of qualitative and quantitative methodologies to facilitate research that is attentive to local contexts and comparable. In the final section, we discuss “meal presentation” or ways to make our research results “appetizing” and *transferable* to a range of stakeholders.

Thus, we aim for this short “cookbook” to serve a dual purpose. On the one hand, we envision it as a jumping-off point for scholars unfamiliar with the vulnerability literature. On the other, we hope our perspective will inspire scholars more familiar with this topic to develop new ways to navigate the tension between locally-specific assessments of vulnerability and attempts at standardization. Our review is not exhaustive. Rather, we focus on recent trends in the vulnerability literature with the intention of catalyzing continued thinking about balancing richness and replicability in future research.

## Background

Before diving into the meat of this “cookbook” we note that despite decades of vulnerability research, there is little consensus regarding the meaning of this concept (10). This is reflected in the variety of terms related to vulnerability including procedural/contextual vulnerability (11), outcome vulnerability (12), structural vulnerability (13), social vulnerability (14), and participatory vulnerability (15). Further complicating interpretations of this work is the fact that there are significant conceptual overlaps between vulnerability and related terms such as exposure, susceptibility, coping, resilience, adaptation, transformation, and sustainability (16, 17). Despite these conceptual overlaps, vulnerability research remains divided along multiple theoretical fracture lines (18). This can make for confusing reading that risks alienating researchers from different disciplines and stakeholders outside academia.

Although reviews of vulnerability research exist (4, 6, 18), we see a space for an updated and succinct summary of this work. To do this, we utilized academic search engines such as Science Direct and Google Scholar with key words such as “vulnerability,” “socio-ecological systems,” “health,” and “well-being” to identify relevant papers. We constructed a matrix with cells for the aim of the paper, methods, definition of vulnerability, theoretical or conceptual models used, domains measured, themes, communication of results, and policy applications. We reviewed more than 50 articles published in the last 15 years focused on vulnerability research allowing us to identify the “recipes,” “ingredients,” “cooking tools,” and “approaches to meal presentation” for our “cookbook.”

### Section 1: “Recipes”

Any theoretical framework for the study of vulnerability should address the complex, non-linear interactions existing between social, cultural, political, ecological, and biophysical processes (19). However, a critique of the concept of vulnerability is that it is so broad, so inclusive, that it becomes a momentous task to consider all the intellectual dimensions involved (20). Therefore, we advocate selecting a theoretical framework that aligns with the strengths and goals of the researcher. This is especially important considering that many papers do not mention the application of a particular theoretical framework (17), even though different framing prioritizes the production of different knowledge and responses (12).

Below we discuss four theoretical perspectives employed in recent empirical and review papers addressing vulnerability, global change, and health. Each paragraph contains an accessible definition, key terms associated with each approach, commonly addressed topics, and applications.

#### Risk-Hazard Approach

The risk-hazard approach examines the impacts of a hazard on an exposed entity (21) and defines vulnerability as a function of exposure, sensitivity, and adaptive capacity (22). Common hazards include famines, floods, drought, seismic events, and technological failures (23). Associated key terms include biophysical, disaster, natural hazards, perturbations, pressure-and-release (PAR) model (24), exposure, risk, and sensitivity. This approach has been used in the development of a range of vulnerability indices (25–27) and widely applied in a number of recent studies [see (9, 26, 28–30)]. It is particularly helpful for engineers, economists, and development professionals in disaster research (6).

#### Political-Ecology Approach

In contrast to the “geocentric,” risk-hazard approach the “anthropocentric,” political-ecology approach (6) argues that hazards are not natural, but rather dependent upon societal factors (31). Examples of socially-conditioned hazards include pollution, poor housing and infrastructure, social fragmentation, and trade liberalization (32). Decision-making power is central to this model (33) and vulnerability can be defined as the risk created by exposures to unequal power relationships and hierarchical social orders (13). Key terms associated with this approach include inequality, injustice, power, marginalization, exploitation, discrimination, structural, and historical factors. Unsurprisingly, this interpretation is of interest to scholars concerned with historic, social, economic, and environmental inequalities (34) from a range of disciplines [see (11, 13, 31, 35, 36)].

#### Resilience Approach

Resilience is defined as the ability of a system to absorb shocks and regenerate after a disturbance. In this school of thought, vulnerability can be defined as “the attributes of persons or groups that enable them to cope with the impact of disturbances” [(37), p. 237]. A key idea is that of “adaptive capacity” or the ability of the exposed entity to mitigate impacts, take advantage of new opportunities, and cope with novel stressors. Associated key terms include agency, systems-thinking, social structure, coping, adaptive capacity, social conditions, thresholds, and persistence. A resilience approach is particularly useful in determining policy-oriented interventions for dealing with uncertainty, future change, and adaptive capacity (38) and is typically utilized by scholars in interdisciplinary programs [see (17, 39–41)].

#### Sustainable Livelihoods Approach

While a resilience model takes a “systems-approach,” a sustainable livelihoods approach starts with a “bottom-up” perspective to understanding how resources are mobilized on a local level (5). A livelihood is defined as the capabilities, assets, and activities necessary to support a means of living (42) and is considered sustainable if it does not undermine the natural resource base (43). In a sustainable livelihoods approach, researchers examine human, social, natural, physical, and financial capitals (44). Similarly, to the previous perspectives, vulnerability can be defined as what may happen to a specific population under conditions of particular risks and hazards. The difference is an emphasis on vulnerability as being *predictive* as it should aid in directing interventions or supporting livelihoods (45). This approach has been used successfully as the basis for development programs and practices (46).

In several recent papers (5, 26, 31, 40, 44, 47, 48), the sustainable livelihoods approach functioned as a bridge between theory and methods by enabling scholars to pinpoint a number of relevant and measurable domains related to the concept of vulnerability. In the next section, we discuss considerations for selecting “ingredients” for a vulnerability assessment and use the five capitals of the sustainable livelihoods as an example of connecting theory to measurable domains.

### Section 2: “Ingredients”

Going from a theoretical framework to deciding what to measure in a vulnerability study can be particularly daunting as the range of potential topics is extremely broad. In Table 1, we use the sustainable livelihoods approach to identify “ingredients” or domains and measurable sub-domains. Table 1 is not a prescriptive list of “ingredients,” but rather an example of how to translate abstract concepts into measurable aspects of vulnerability research.

**Table 1:** “Ingredients” for consideration: livelihood capitals, domains, and sub-domains.

**Livelihood capitals**	**Domains**	**Measurable sub-domains**
Human capital	Education, knowledge, skills, capacity to work, health, nutrition, capacity to adapt	Highest educational qualification, dependency ratio, occupational training, perceived health, costs for medical treatment
Social capital	Social connections and networks, religious/cultural beliefs, local knowledge, participation in decision-making	Memberships in local, national, and international organizations, expenditures on social events, traditional ecological knowledge
Natural capital	Biodiversity, land, water, aquatic resources, trees and forest products, wild foods and fibers, ecosystem services	Quality of the land possessed, area under cultivation, area of woodland or fruit trees, access to water for irrigation
Physical capital	Infrastructure, tools, and technology	Type and expenditure for housing, distance to nearest road, tools and equipment for production, traditional technology, shelter, water supply and sanitation, energy, communications
Financial capital	Savings, credit, debt, remittances, pensions, wages	Gross household annual income, household savings, and ownership of livestock

This process can be extended to the risk-hazard, political-ecology, and resilience approaches through a review of each body of literature and highlights two take-away messages. The first message is the ‘T' of *transparency*, or being clear about the research process (49), especially in regard to the framing that leads to the selection of particular indicators. The second message relates to the ‘T' of *transferability*, or the study's potential to be valuable across contexts and situations (49). One way to do this is to question whether the values and ideas of the primary investigators are valid or relevant in a particular field-site by eliciting the voices of the people with whom you work before moving forward with measurement (50). With this in mind, in the next section, we discuss “cooking tools,” or qualitative and quantitative methods, which can yield data that is both “place-based” and amenable to comparison and replicability.

### Section 3: “Cooking Tools”

In the words of Veland et al. (11) researchers need methods enabling us to “see with both eyes” (p. 316) to capture what it means to be vulnerable from a local and scientific perspective. We present Table 2 as a drawer of “cooking tools” (qualitative, quantitative, and mixed methodological approaches), presented with key terms, related methods, and references, that we believe can help scholars select approaches that capture both angles.

**Table 2:** “Cooking tools” for consideration: qualitative, quantitative, and mixed methods.

**Approach**	**Key terms**	**Methods/indices**	**References**
Qualitative	Place-based, context-dependent, bottom-up, multi-stakeholder, participatory, dialogue-oriented, reflexive	Participant observation, interviews, focus group discussions, community workshops, future story-lines, biographies, photovoice	(11, 15, 20, 51–55)
Quantitative	Comparative, standardized, indices^*^, scales, repeatable	Survey, census, livelihood vulnerability index, index of vulnerability, social vulnerability index, hunger, and climate vulnerability index	(8, 10, 56–61)
Mixed	Hybrid, integrated, multi-method, equal status design, mixed priority design	Socioecological matrices, integrated assessment map, rapid rural appraisal, participatory action research, citizen science	(9, 56, 62–64)

* see Hinkel (7), Barnett et al. (65) for caution in the application of indices.

While we separate Table 2 into qualitative, quantitative, and mixed method categories, many of the studies found in each of the groupings utilize *triangulation*, or the use of multiple sources of data and conceptual frameworks (49). One example is rapid rural appraisals, which utilize small, multi-disciplinary research teams who employ mixed methods “in an intensive, iterative, and expeditious manner” [(53), p. 1069]. For example, Berrang-Ford et al. (53) used semi-structured interviews, key informant interviews, future story-lines, biographies, and photovoice to understand vulnerability to climate change among the Batwa of Uganda. Using the risk-hazard approach and basic quantitative analyses, they identified common themes in their qualitative data that shed light on exposure, sensitivity, adaptive capacity, and related health outcomes in this context. They found that climate change interacted with socioeconomic factors to increases risk for water and food insecurity, exposure to disease vectors, and weather-related stressors (53).

In the Berrang-Ford et al. (53) study, participant voices were integrated into the research. However, Fazey et al. (15) advocate for even deeper integration of community members in the research process with the aim of building local capacity and enhancing equity to reduce vulnerability. For example, Fazey et al. (15) worked collaboratively with local organizations in the Solomon Islands to train members to conduct vulnerability assessments. Periods of formal reflection were facilitated among primary researchers, research assistants, and community members to encourage engagement with challenges such as fluctuating global markets and a growing population. This highly participatory process facilitated co-learning between researchers and community members, generated high quality information amenable to academic analysis, and increased the confidence of local people to address issues with food insecurity, health, and resource availability.

By integrating community members into research design and data collection, deeply participatory research can keep the results of the study in the hands of local people, allowing them to use it for communications, political lobbying, or further investigation of the challenges they face. In the final section, we expand on the value of transferability (italicized), by discussing ideas for creatively communicating research results to a multi-stakeholder audience to enhance the impacts of vulnerability investigations.

### Section 4: “Meal Presentation”

While our “chefs” may have used tried and true recipes, with delicious ingredients, and state-of-the-art cooking tools, if the meal does not look appetizing, no one will want to eat it. What we mean is that if researchers do not think about presenting their results in an accessible format, the larger body of stakeholders who can benefit from this work will not consume the results.

With this in mind, a key challenge for successful *transferability*, or successful uptake of the work, is to communicate results *with*—not *to*—stakeholders (20). This requires scholars to think about how they can align their take-home-messages with local norms (54). For example, Veland et al. (11) used ceremony, storytelling, talk of dreaming, and the *logic of country* to communicate their results to the Aboriginal Australian peoples with whom they worked. Indeed, jargon-free language and alternative formatting, such as storytelling, graphic displays, and mapping, can make research results accessible to a range of stakeholders. These stakeholders can then use the information for educational initiatives, mitigation programs, and even humanitarian relief distribution programs (66).

Such efforts can be “low tech and low cost.” In their work in rural villages in the Philippines, Gaillard et al. (67) helped residents make 3D town maps with dough and plywood to identify the most vulnerable locations and homes. Data from these maps were entered into geographic information system databases and digitized for use by local scientists and governments, integrating local and scientific knowledge, as well as bottom-up and top-down disaster risk management (67). Finally, advances in communication and information technologies, such as innovations in the use of tablets, smartphones, and “smart applications” can close the gaps between citizens and scientists, making research more collaborative, transparent, and efficient (68).

In summary, there are many creative ways to conduct vulnerability research that is theoretically-informed, “place-based,” and amenable to comparison. A final example that portrays how a scholar can use the “cookbook” approach presented here is Tallman's (56) Index of Vulnerability (IoV). This quantitative index was inspired by Leatherman's (34) articulation of a “space of vulnerability,” which used a political-ecological approach to examine the intersection of poverty, hunger, nutrition, and health. Tallman (56) used this “recipe” in conjunction with the “cooking tools” of participant observation, interviews, and focus group discussions with Awajún community members in the Peruvian Amazon to identify the “ingredients” of food insecurity, water insecurity, social support, social status, and healthcare access as important to understanding vulnerability in this context. Scores on the IoV increase for each life domain where the individual falls into a “high risk” category. This approach makes the IoV standardized but also malleable to local contexts, as scholars can choose which measure of each life domain is most appropriate for their study population. In research among the Awajún, the IoV was associated with measures of stress, perceived health, energetic reserves, depressive, and somatic symptoms (56). These results were shared with stakeholders through an interdisciplinary conference bringing together scholars, government officials, indigenous leaders, and non-governmental organization representatives to discuss vulnerability among indigenous Amazonian peoples. What emerged from this event was that if stakeholders feel they own the process (i.e., participating in design and data collection), information (i.e., seeing their views represented), and materials (i.e., being involved in development and diffusion of key messages and visuals), they will be more likely to share the findings widely, creating a ripple of impacts in different arenas.

## Conclusion

In this perspective article, we aimed to mitigate the, at times, overwhelming complexity of vulnerability scholarship by increasing the accessibility of this work using the metaphor of a “cookbook.” Specifically, there is an abundance of confusing and overlapping terminology in vulnerability research, which necessitates that researchers are *transparent* about the meanings and usage of theoretical frameworks, methods, and indicators in their work (7, 69). Additionally, there are a number of studies that utilize deeply participatory methods but lack explicit theoretical models—and vice versa (15). *Triangulation* can be among theory and methods, data and investigators (14) or among sources, methods, and other investigations (53). This allows “different facets of problems to be explored, increases scope, deepens understanding, and encourages consistent (re) interpretation” [(49), p. 843], which can yield vulnerability research that is “good science” from a methodological and ethical perspective. In conclusion, vulnerable people and places are often excluded from decision-making, power, and resources (4), despite the fact that local knowledge can provide key insights into understanding vulnerability and adaptation (54). Thus, we need to think about ways that our research can be *transferable* to multiple stakeholders by accommodating alternative research questions and methods of inquiry (11) and creating effective relationships for knowledge sharing. Ultimately, we hope that attention to these issues will yield research that is rich, replicable, and amenable to facilitating social justice and human dignity for the most vulnerable among us.

## Author Contributions

PT led the writing of this manuscript. AV-V, GS-M, GL, AR-P, LB-V, SH, and VP-S all contributed to the development of the ideas within the manuscript and substantially contributed to revising it throughout the writing process.

### Conflict of Interest

The authors declare that the research was conducted in the absence of any commercial or financial relationships that could be construed as a potential conflict of interest.
